# Stochastic Termination of Spiral Wave Dynamics in Cardiac Tissue

**DOI:** 10.3389/fnetp.2022.809532

**Published:** 2022-01-26

**Authors:** Wouter-Jan Rappel, David E. Krummen, Tina Baykaner, Junaid Zaman, Alan Donsky, Vijay Swarup, John M. Miller, Sanjiv M. Narayan

**Affiliations:** ^1^ Department of Physics, University of California, San Diego, La Jolla, CA, United States; ^2^ Department of Medicine, University of California, San Diego, La Jolla, CA, United States; ^3^ Department of Medicine and Cardiovascular Institute, Stanford University, Palo Alto, CA, United States; ^4^ Department of Medicine, Division of Cardiology, University of Southern California, Los Angeles, CA, United States; ^5^ Baylor University, Dallas, TX, United States; ^6^ Arizona Heart Rhythm Institute, Phoenix, AZ, United States; ^7^ Krannert Institute, Indiana University, Indianapolis, IN, United States

**Keywords:** cardiac arrhythmia, ablation, modeling, spiral wave, atrial fibrillation

## Abstract

Rotating spiral waves are self-organized features in spatially extended excitable media and may play an important role in cardiac arrhythmias including atrial fibrillation (AF). In homogeneous media, spiral wave dynamics are perpetuated through spiral wave breakup, leading to the continuous birth and death of spiral waves, but have a finite probability of termination. In non-homogeneous media, however, heterogeneities can act as anchoring sources that result in sustained spiral wave activity. It is thus unclear how and if AF may terminate following the removal of putative spiral wave sources in patients. Here, we address this question using computer simulations in which a stable spiral wave is trapped by an heterogeneity and is surrounded by spiral wave breakup. We show that, following ablation of spatial heterogeneity to render that region of the medium unexcitable, termination of spiral wave dynamics is stochastic and Poisson-distributed. Furthermore, we show that the dynamics can be accurately described by a master equation using birth and death rates. To validate these predictions *in vivo*, we mapped spiral wave activity in patients with AF and targeted the locations of spiral wave sources using radiofrequency ablation. Targeted ablation was indeed able to terminate AF, but only after a variable delay of up to several minutes. Furthermore, and consistent with numerical simulations, termination was not accompanied by gradual temporal or spatial organization. Our results suggest that spiral wave sources and tissue heterogeneities play a critical role in the maintenance of AF and that the removal of sources results in spiral wave dynamics with a finite termination time, which could have important clinical implications.

## Introduction

Spiral waves are examples of self-organized activity in spatially extended excitable media and have been studied in a variety of biological and non-biological systems (Jakubith et al., 1990; Lechleiter et al., 1991; Huang et al., 2004; Sawai et al., 2005; Beta et al., 2006; Yu et al., 2012). In cardiac tissue, they form when activation fronts break and reenter unexcited tissue (Karma, 2013). Spiral waves are associated with cardiac arrhythmias and may be responsible for the initiation and maintenance of ventricular and atrial fibrillation (AF). AF is the most common arrhythmia that currently affects over 30 million people world-wide and leads to an increase in stroke, heart failure and mortality (Chugh et al., 2014). The most frequently applied therapeutic treatment option is ablation, which surgically destroys cardiac tissue. Specifically, ablation is used to electrically isolate the pulmonary veins, which can harbor AF initiating triggers (Haïssaguerre et al., 1998). This treatment, however, yields suboptimal results (Verma et al., 2015), which has led to attempts to detect spiral waves through patient-specific computational models (Prakosa et al., 2018; Boyle et al., 2019) and visualization efforts (Baykaner et al., 2018) to improve therapy.

A single spiral wave in the heart can result in activation with a higher frequency than normal sinus rhythm and is associated with tachycardias. Computational studies of spiral wave activity in cardiac tissue have shown that a spiral wave can break up into multiple spiral waves through a variety of mechanisms, resulting in disorganized activity and fibrillation-like dynamics (Moe et al., 1964; Winfree, 1987; Fenton et al., 2002). The tip of these spiral waves, where the activation and repolarization waves meet, represents a phase singularity, can be determined using a variety of algorithms (Bray et al., 2001; Iyer and Gray, 2001; Fenton et al., 2002). Experimentally, spiral waves, their break up and their singularities were demonstrated during cardiac fibrillation in isolated tissue and in several animal models (Davidenko et al., 1992; Gray et al., 1998; Skanes et al., 1998; Witkowski et al., 1998; Zaitsev et al., 2003; Cherry and Fenton, 2008; Christoph et al., 2018). In humans, spiral waves have been shown in the fibrillating atria of explanted hearts using optical mapping, and show agreement with the results from multi-electrode catheter systems that are used clinically (Hansen et al., 2018). Several systems are now used in patients that are inserted into one or both atria to record electrical signals from large spatial areas and reveal spiral wave activity in AF patients (Narayan et al., 2012a; Bellmann et al., 2019; Willems et al., 2019; Choudry et al., 2020).

The break up of spiral waves is a stochastic process during which spiral waves are born and die in a random fashion. If the tissue is homogeneous, the distribution of spiral wave tips would also be homogeneous and several quantities can be computed using techniques from statistical physics (Vidmar and Rappel, 2019). For example, using the birth and death rates of the spiral tips, one can compute the mean termination time *τ* and can show that it depends exponentially on tissue size (Aron et al., 2019; Vidmar and Rappel, 2019). This is consistent with the critical mass hypothesis, which states that fibrillation with a characteristic wavelength requires a minimal organ size (Garrey, 1914; Byrd et al., 2005; Qu, 2006). Inhomogeneous tissue, however, can create regions that are acting as sources for spiral waves. For example, simulations have shown that fibrotic tissue, due to its slow conduction and reduced excitability, can harbor reentry while the surrounding tissue exhibits spiral wave breakup (McDowell et al., 2015) while localized regions with fast propagation velocity are able to sustain spiral wave generation (Zykov et al., 2017). The role of these sources in AF was demonstrated in clinical work, which showed that targeted ablation of rotational sources, during which tissue is destroyed, was able to terminate AF (Narayan et al., 2012b).

Previous studies did not determine how AF terminated following targeted ablation of spiral wave sources and the precise route to termination is therefore currently poorly understood. Destroying tissue that is responsible for the maintenance of spiral wave dynamics may not eliminate the arrhythmia immediately (Narayan et al., 2012a; Bellmann et al., 2019; Willems et al., 2019; Choudry et al., 2020). To determine how spiral wave dynamics are affected by the removal of sources, we studied AF termination using both simulations and in AF patients in which ablation is only used to target spiral wave sources. In our simulations we show that the targeted removal of spiral wave sources leaves AF-like dynamics in the remainder of the tissue. We also show that the resulting dynamics is stochastic with termination times that are Poisson-like distributed and that can be computed using the birth and death rates of spiral tips. We then examined the spatiotemporal dynamics of human AF in patients by recording electrical signals from multielectrode arrays inserted in both atria. The resulting activation maps were used to guide targeted therapy at spiral wave sources. The organization of AF was measured using several quantitative indices and, in patients in whom this approach terminated AF and with a temporal resolution of 1 min, termination was abrupt rather than progressive and was often delayed by minutes. Together, these results suggest that, following the removal of spiral wave sources, the termination of AF is a stochastic process.

## Methods

### Computational Studies

Spatially extended simulation studies were carried out using an electrophysiological model based on Luo-Rudy membrane kinetics (Luo and Rudy, 1991) with parameters modified to represent atrial cellular properties (see Supplementary Material) (Li et al., 2001; Virag et al., 2002). Additional simulations were carried out using the Fenton-Karma model (Fenton and Karma, 1998), as detailed in the Supplementary Material. Simulations were performed in a square domain of area *A* with non-conductive boundaries. A tissue inhomogeneity, representing a region of depressed excitability, was introduced by modifying the parameters within a central disk-shaped region with radius *R*. Virtual ablation lesions were introduced by replacing this inhomogeneity by an inexcitable region. Non-conducting boundary conditions of the resulting curved boundary were incorporated using the phase field method (Fenton et al., 2005). After ablation, the total length of the non-conductive boundary was $L=4\sqrt{A}+2\pi R$ while the net area was $A_{net}=A-\pi R^{2}$ .

### Clinical Mapping and Ablation

We studied 31 patients with early atrial fibrillation (paroxysmal AF) undergoing their first ablation for routine indications at five medical centers. Patient inclusion and exclusion criteria and population details are presented in Supplementary Table S1. Written, informed consent was granted for all patients prior to enrollment in National Institutes of Health protocols (NCT01248156), which were approved by the Institutional Review Board of each center.

Electrograms were recorded from multielectrode basket arrays inserted in the atria (Supplementary Figure S1). Activation times were annotated within 4s intervals and used to determine an activation front. From the activation fronts we determined a wavefront field (WFF) describing conduction propagation using stream lines. These streamlines can be used to determine rotational activity within the mapped domain using the vector curl of the flow velocity (Vidmar et al., 2016; Bhatia et al., 2020). Specifically, the rotational activity can be quantified by the vorticity Ω , which takes on values between -1 and +1, with negative/positive values corresponding to counterclockwise/clockwise rotating spiral waves.

The level of synchrony between electrode pairs was calculated by computing the synchronization number *γ*, which quantifies the extent to which that pair displays synchronous activation sequences and which varies between 0 (asynchronous) and 1 (perfect synchrony). A global measure of spatio-temporal organization for a given episode was subsequently determined by computing the mean synchronization number ‹γ› across all pairs of electrodes (Vidmar et al., 2015). In addition, we computed phase maps using standard methodologies (Narayan et al., 2012a). From these phase maps, we quantified the number of phase singularities (PSs), representing spiral wave tips (Bray et al., 2001). Both the number of phase singularities and the mean synchronization number were computed 3 min preceding (T-3), 1 min preceding (T-1), and immediately preceding AF termination (T). Targeted ablation was guided by WFF and applied to zones of high rotational activity under a specific protocol (clinicaltrials.gov: NCT03702244). No other ablation, including pulmonary vein isolation, was performed. The extent of ablation (duration 17.2 + 8.2 min) was lower than would be typical for pulmonary vein isolation (in the range of 31–37 min) (Oral et al., 2006; Sørensen et al., 2021).

### Statistics

Continuous data are represented as mean ± standard deviation. Normality was evaluated using the Kolmogorov-Smirnov test. Comparisons between 2 groups were made with Student’s t-tests if normally distributed or, if not normally distributed, evaluated with the Mann-Whitney *U* test. Nominal values are expressed as n (%) and compared with chi-square tests or the Fisher’s exact test for comparisons when expected cell frequency was <5. A probability of <0.05 was considered statistically significant.

## Results

### Simulation Results

**FIGURE 1:**
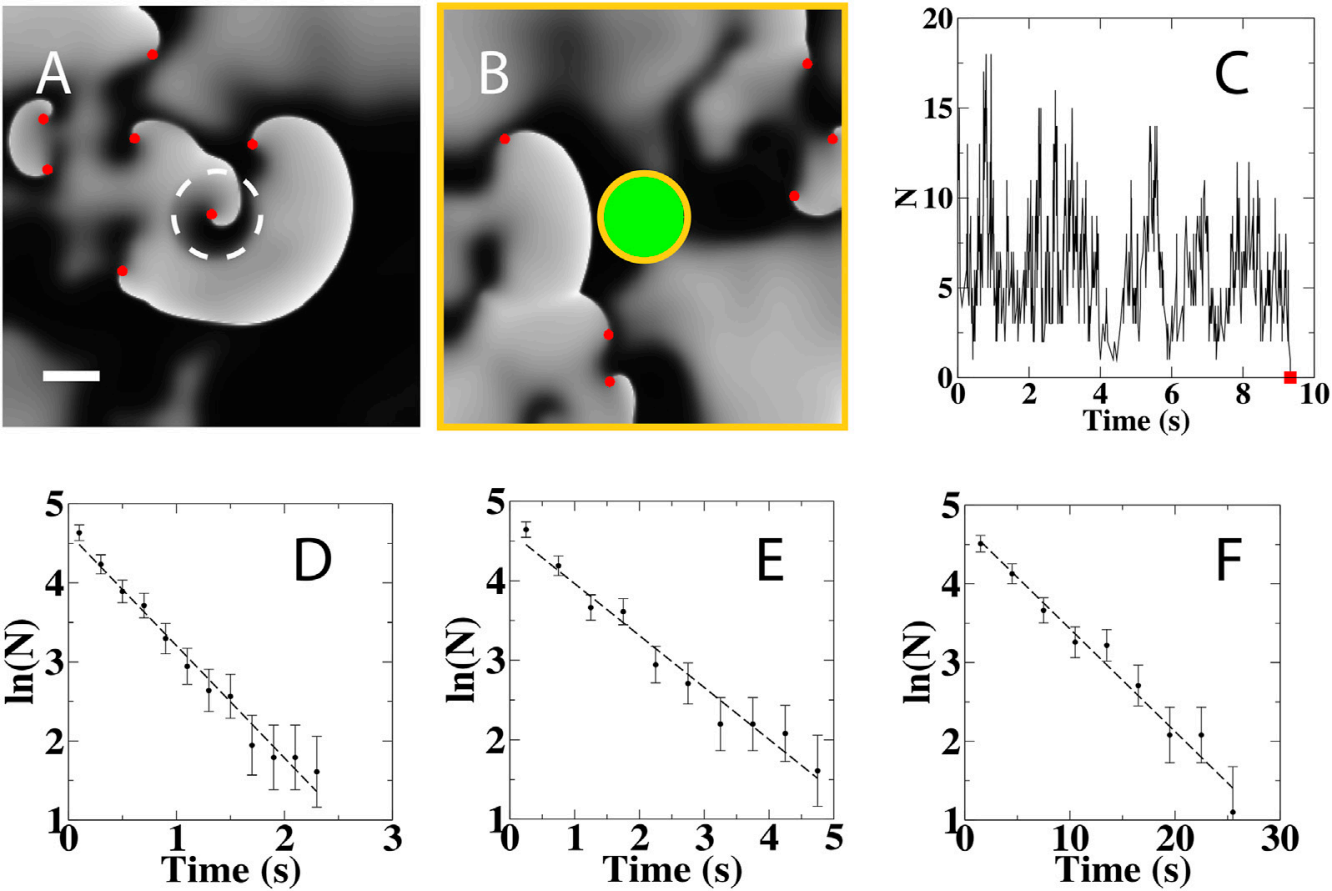
Computational Simulations of Delayed AF Termination and Numerical Analyses. **(A)** Snapshot of a simulation in a 7.5 × 7.5 cm domain showing the activation pattern during spiral wave break up, with white (black) corresponding to activation (recovered) tissue and with spiral wave tips indicated by red symbols. A central portion of the computational domain, indicated by the white circle with radius of 0.75 cm, exhibits reduced excitability resulting in a stable spiral wave source. Scale bar: 1 cm. **(B)** Snapshot of the activation following ablation of the heterogeneous circular region in A, indicated in green, showing the removal of the spiral wave from the central region. All non-conducting boundaries are shown as orange lines. **(C)** Number of spiral tips as a function of time during a typical simulation with a domain size of 100 cm^2^. This number fluctuates and can reach zero (red square), resulting in the termination of SDC. **(D–F)**, Distribution of termination times, computed using 400 independent simulations, for a domain size of 39 cm^2^ **(D)**, 56.25 cm^2^ **(E)**, and 100 cm^2^ **(F)**. The dashed line is an exponential fit to the distribution.

To study the mechanisms by which spiral wave dynamics in AF may be impacted by elimination of sources, we simulated spiral wave reentry in a 2D cardiac model linking the membrane potential and ionic currents (Supplementary Material). Electrophysiological parameter values were chosen such that spiral waves in homogeneous tissue (i.e., in the absence of a heterogeneity) are dynamically unstable and exhibit continuous break up. To model a localized source, we introduced a disk-shaped heterogeneity (with radius R = 0.75 cm) of reduced excitability in the center of the domain (white dashed line, Figure 1A), resulting in a meandering spiral wave surrounded by spiral wave break up that maintained fibrillation-like activation indefinitely (Figure 1A). This spiral wave was stable and remained in the heterogeneous zone indefinitely (Supplementary Figure S2). Numerical ablation of the localized source, through the creation of a non-conducting disk-like zone, resulted in the removal of the source (Figure 1B). The remainder of the tissue continued to exhibit spiral wave break down and the dynamics, immediately following the introduction of the virtual lesion, depended on the state of the system at the time point of ablation. This dynamics was characterized by stochastic creation and annihilation of spiral waves and, since the localized source was removed, had a finite lifetime.

By computing the locations of the spiral wave tips, we recorded the number of remaining spiral tips as a function of time. This number changed due to the interaction of the spiral waves with the non-conducting boundaries and with each other. Specifically, the number decreased by one through the annihilation of single tips that collided with non-conducting boundaries and decreased by two due to tip collisions. Furthermore, the number increased by one through tip generation near non-conducting boundaries and by two due to wave break. The tip number fluctuated around a constant average value due to spiral wave dynamics and was extinguished when it fell to zero, corresponding to spontaneous self-termination (Figure 1C).

To quantify the termination process, we repeated simulations starting with distinct initial conditions (Methods) and determined the termination times following virtual ablation for different domain sizes. The distribution of these termination times, computed using 400 simulations, was fitted well with an exponential for all domains sizes with a rate *λ* (Figures 1C–E). This indicates that termination is a stochastic event that can be characterized by a Poisson process, with an average termination time, *τ*, given by the inverse of λ: *τ* = 1/λ. The values for *τ* obtained by fitting the distribution agree well with the termination time obtained from the simulations (Table 1). Simulations also showed that the mean termination time depends on the geometry of the domain and varies exponentially with the size of the net domain *A*_*net*_ (Vidmar and Rappel, 2019) (Supplementary Figure S3). Note that the values for *τ* are smaller than for the homogeneous case without the virtual ablation lesion presented in an earlier study (Vidmar and Rappel, 2019). This is to be expected since the ablation lesion reduces the size of the computational domain and introduces an additional non-conducting boundary. Qualitatively similar results were obtained for a different electrophysiological model, indicating that the results are model independent (Supplementary Figures S3, S4; Supplementary Table S2).

**TABLE 1:** Termination times computed using simulations on square domains, obtained from fits to the distribution, and determined using the transition rate matrix.

Domain size *A* (cm^2^)	τ (simulations) (s)	1/λ (Poisson) (s)	τ (matrix) (s)
39	0.73	0.7	0.7
56.25	1.43	1.5	1.4
100	7.6	7.7	7.5
156.25	68	72	68

**FIGURE 2:**
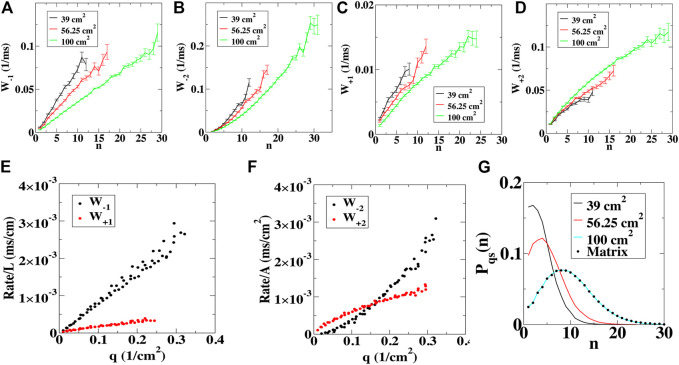
Transition rates computed using numerical simulations. **(A–D)**, The birth and death rates for the removal of a single (n→n−1 **(A)**) and a pair of spiral wave tips (n→n−2 **(B)**), along with the birth rates for the creation of a single (n→n+1 **(C)**) and a pair of spiral wave tips (n→n+2 **(D)**) computed in domains of various sizes. Error bars represent standard deviation. **(E)**, The birth and death rates for a single tip, W_±1_, normalized by the perimeter of the domain, as a function of the density of tips, *q = n/A*_*net*_. **(F)**, The birth and death rates for a pair of tips, W_±2_, normalized by the area of the domain, as a function of the density of tips. **(G)**, The quasi-stationary distribution for different domain sizes computed using the simulations (lines) and computed using the transition matrix (symbols).

We also computed the birth and death rates as a function of the number of tips *n* by quantifying the number of transitions per time interval (Supplementary Materials). The rates for the annihilation and creation of a single tip, *W*_*-1*_ *(n)* and *W*_*+1*_ *(n)*, show an approximately linear dependence on the number of tips (Figures 2A,B). The rates for the annihilation and creation of two tips, *W*_*-2*_ *(n)* and *W*_*+2*_ *(n)*, on the other hand, show a more complex dependence on *n* (Figures 2C,D). For all domains, however, we found that rescaling the *±1* rates by the total length *L* of the non-conducting boundary present in the computational domain collapsed all rates onto a single curve when plotted as a function of the density of tips *q = n/A*_*net*_ (Figure 2E). Furthermore, we found that rescaling the *±2* rates by the net area of the computational domain also produced a single rate curve (Figure 2F). In other words, these rates only depend on the density of tips, indicating that the tips are well-mixed.

Using the birth and death rates, we can formulate a master equation, which describes the probability *p(n,t)* of having *n* spiral tips at time *t*:

$$\frac{dP\left(n,t\right)}{dt}=\underset{r}{\sum }\left[W_{r}\left(n-r\right)P\left(n-r,t\right)-W_{r}\left(n\right)P\left(n,t\right)\right]$$

where the sum is over *r = ±1* and *r = ±2*. As boundary conditions, we take *W*_*r*_ *(0) = 0*, corresponding to an absorbing boundary at *n = 0*, and *W*_*-2*_ *(1) = 0*, since a single tip cannot collide with another tip. It is not possible to obtain an analytical solution for this equation. However, since for small *n* the birth rates are larger than the death rates while for large *n* the opposite holds true, a metastable state exists with a corresponding quasi-stationary distribution *P*_*qs*_(*n,t*) (Assaf and Meerson, 2010). It is possible to compute this quasi-stationary distribution, using the transition matrix constructed from the transition rates (symbols Figure 2G; see Supplementary Material). This distribution agrees well with the distribution obtained from our direct simulations (lines Figure 2G; Supplementary Figure S4F) and the mean number of tips corresponds approximately to the peak of the distribution. Termination corresponds to a large fluctuation away from this mean number and the mean termination time *τ* can also be computed using the transition matrix (Assaf and Meerson, 2010; Vidmar and Rappel, 2019). The resulting values agree well with the values obtained using either the direct simulations or the exponential fit in Figure 1 (Table 1; Supplementary Table S2).

### Patient Results

**FIGURE 3:**
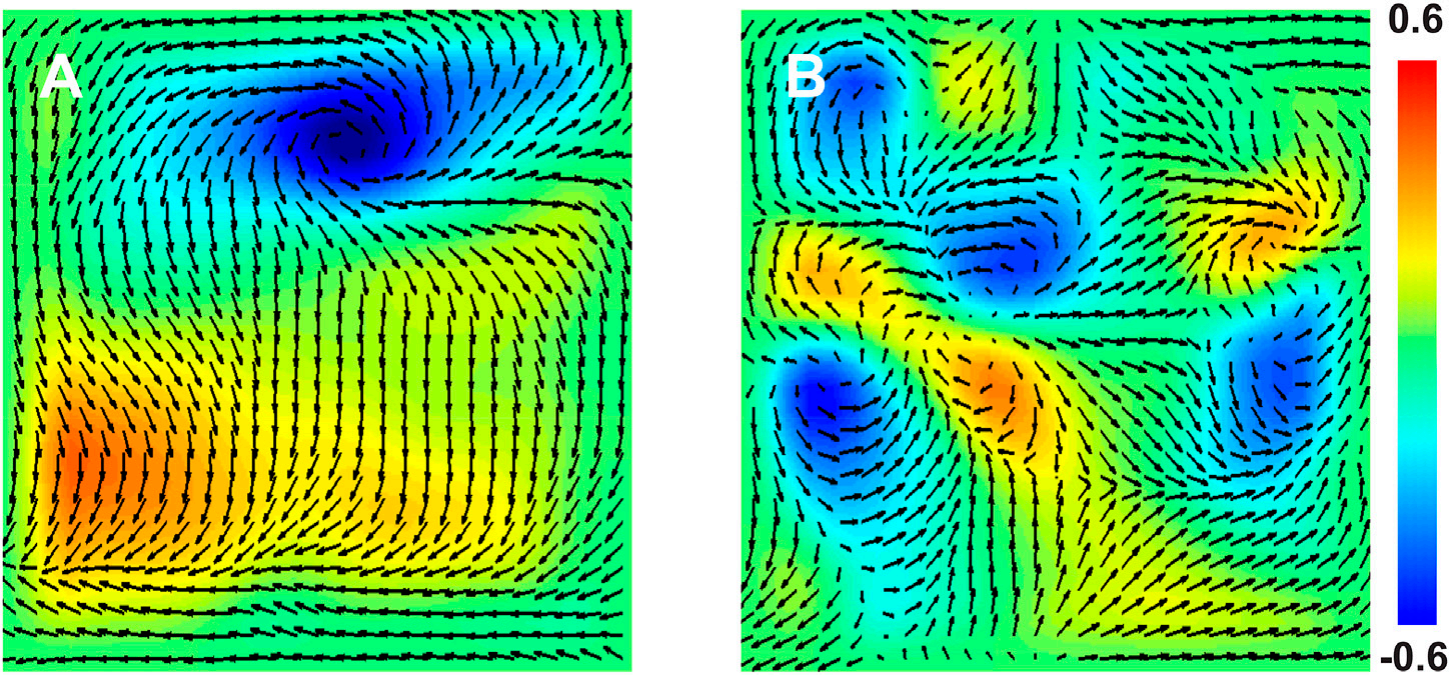
Electrical Organization of Atrial Fibrillation. **(A,B)**: Snapshots of the WFF flow field (arrows) along with a color map of the vorticity during AF in a 45 years old male patient **(A)** and a 59 year old male patient **(B)**. The value of the vorticity can range from -1, for counterclockwise rotating spiral waves, to +1, for clockwise rotating spiral waves.

**FIGURE 4:**
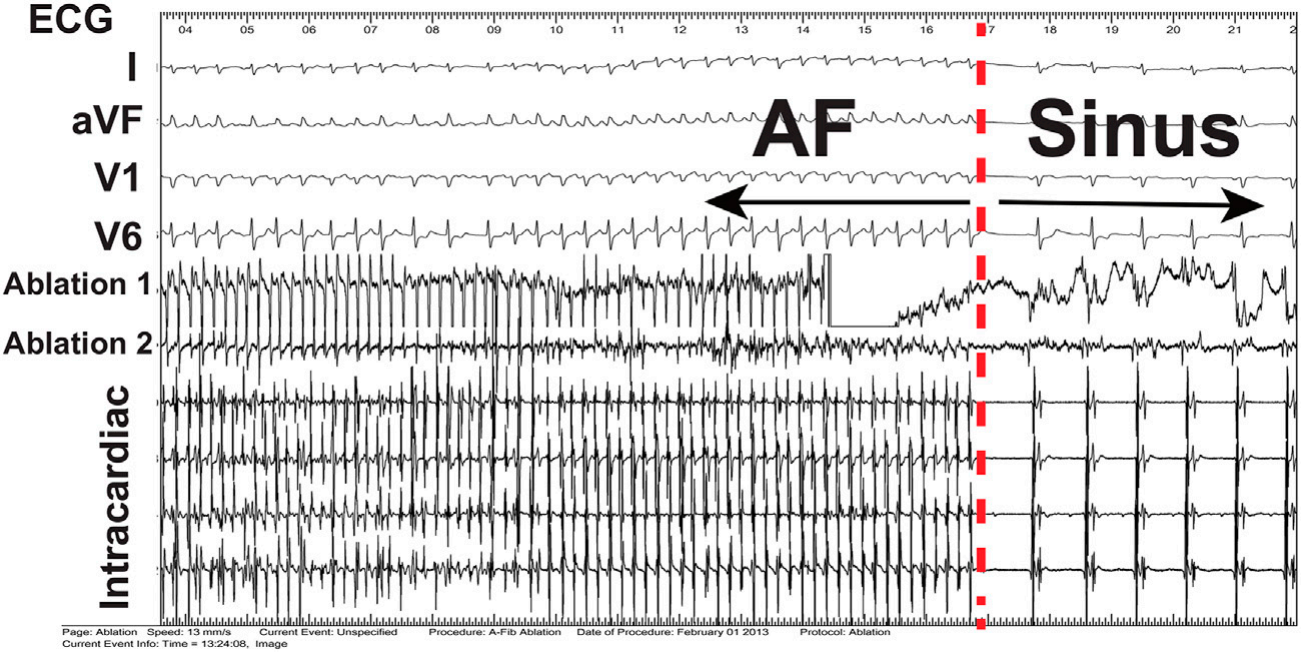
Electrograms during termination. Electrograms from body surface (ECG) and inside the heart (intracardiac) showing termination of AF, indicated by the dashed red line, to sinus rhythm following ablation of spiral wave sources in a 45 year old male patient.

The locations of spiral wave sources in patients were determined by constructing WFF maps (Methods). Examples of these maps, which represent the temporally averaged spatial organization during the 4 s interval, are presented in Figures 3A,B. The maps revealed spatial organization in patients, with clear locations of large and small values of the vorticity as indicated by the color scale, corresponding to rotational sources. We then applied targeted ablation to each source (2.5 ± 1.2 overall with 1.6 ± 0.8 in left atrium and 0.9 ± 0.8 in right atrium). No other ablation lesions, including attempts to isolate the pulmonary veins, were carried out. We found that this targeted ablation was able to terminate AF in 83.9% (26/31) patients, as seen from the patient’s ECG or the intracardiac electrodes (Figure 4). Further clinical details, including long-term freedom of AF, are provided in Supplementary Material (Supplementary Figure S5).

### Spiral Wave Dynamics in Patients

**FIGURE 5:**
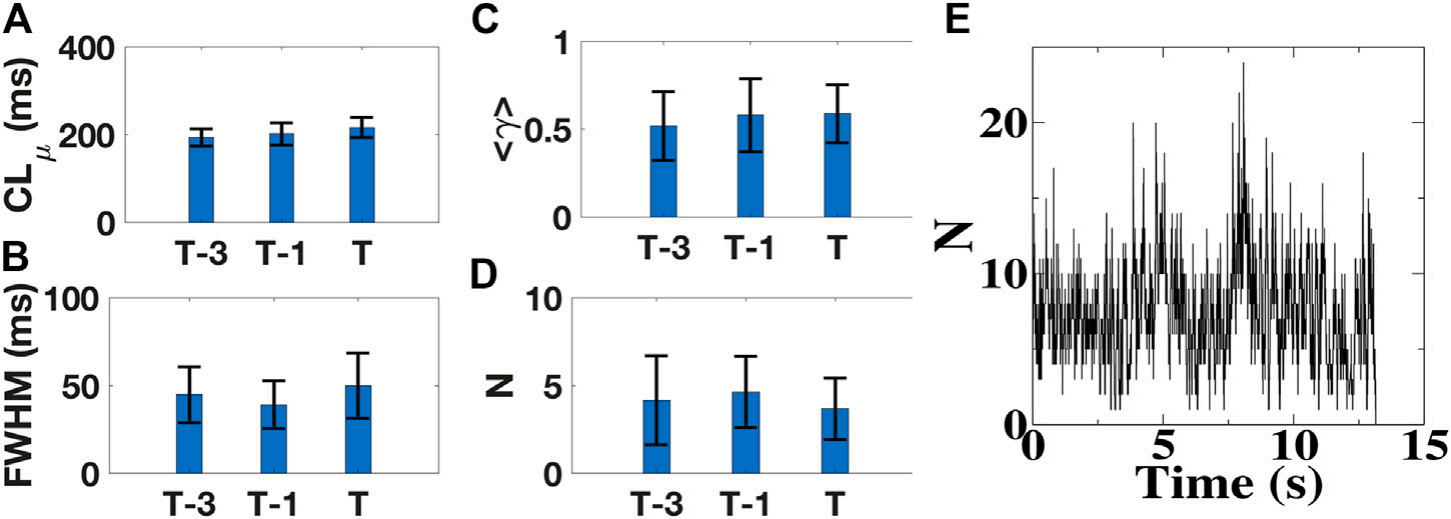
Dynamics of AF termination. **(A–D)**, Mean cycle length CL_μ_ **(A)**, corresponding full width at half-maximum height (FWHM) **(B)**, the average synchronization index <*γ*> **(C)**, and the mean number of tips **(D)** in patients with AF, measured 3 min (T-3), 1min (T-1), and immediately preceding AF termination (T), averaged over all patients. For all quantities there is no significant increase or decrease in successive intervals. **(E)**, Number of tips as a function of time for a 64 year old male patient in which AF converts to sinus rhythm.

Instead of immediately reverting to an organized rhythm, we found that AF terminated following a delay of tens of seconds to minutes (1.2 ± 0.8 min) after elimination of organized AF sources. To determine whether this route to termination was accompanied by a gradual increase in temporal organization, we determined cycle lengths (CLs), defined as the time between two consecutive activations, at each electrode as a function of time. We fitted the resulting distribution of CLs for each patient to a Gaussian function and quantified the full width at half-maximum height (FWHM) and the mean cycle length, CL_μ_ (Supplementary Figure S6). When computed in both atria during 4s intervals at 3 min preceding (T-3), 1 min preceding (T-1), and immediately preceding AF termination (T) (Methods), CL_μ_ increased from 193 ± 20 m, to 201 ± 26 m and 216 ± 23 m, respectively (Figure 5A) but with distributions in successive intervals that did not differ significantly (*p* > 0.08). Overall, patient-averaged FWHM varied from 45 ± 16 m, to 39 ± 14 m and 50 ± 19 m at these timepoints, with distributions that again did not differ significantly (*p* > 0.06; Figure 5B). These results suggest that, examined at a timescale of 1 min, termination was not preceded by a gradual increase in temporal organization.

To quantify spatial organization of AF preceding termination, we determined the mean synchronization number <*γ*> between all electrode pairs (Methods). Low values of <*γ*> correspond to little spatial organization while high values indicate large synchronous regions (Vidmar et al., 2015). For the 4s intervals at 3 min, 1 min, and immediately preceding AF termination, we found that patient-averaged <*γ*> was largely unchanged over time, ranging from 0.52, to 0.59 and 0.58 (Figure 5C; *p* > 0.26). Thus, spatial organization remained relatively constant and did not increase preceding termination at the 1 min temporal resolution.

To further quantify the spatio-temporal organization, we also analyzed phase maps and computed the mean number of spiral wave tips per 4 s interval (Narayan et al., 2012a; Pandit and Jalife, 2013). This analysis showed that this quantity, again averaged over patients, remained near constant for the three intervals, followed by a sudden decrease to zero at termination: 4.2 ± 2.6 for interval T-3, 4.6 ± 2.0 for T-1 and 3.7 ± 1.8 for T (*p* > 0.2), (Figure 5D). When examined during an interval preceding termination, the number of tips fluctuated and AF terminated abruptly when this fluctuation resulted in the absence of any tip (Figure 5E). Taken together, these clinical results are consistent with our numerical studies: AF self-terminates following the removal of sources using targeted ablation in an abrupt rather than progressive manner.

## Discussion

Our simulation results show that elimination of spiral wave sources is able to terminate spiral wave dynamics after a variable delay. This route to termination was demonstrated in computational simulations in which a stable spiral wave is surrounded by unstable spiral wave break up. After removal of the spiral wave source, residual spiral wave activity continued until all spiral wave tips were eliminated. This residual spiral wave activity is characterized by the continuous annihilation and creation of spiral waves through birth and death processes. This creation and annihilation process, which is inherent to excitable media, results in a meta-stable distribution for the number of spiral wave tips that can be determined using the birth and death rates. The number of tips has a non-zero probability to fall to zero, corresponding to spiral wave termination. The distribution of resulting termination times is exponential, indicating that the termination process is stochastic. These results are in agreement with a recent study which showed that phase singularities, corresponding to spiral wave tips, are exponentially distributed in simulations, human AF and animal models (Dharmaprani et al., 2019). Importantly, we carried out simulations using different models (the Luo-Rudy and Fenton-Karma model), indicating that our computational results are not specific to the details of the studied model and may apply to a variety of electrophysiological models (Vidmar and Rappel, 2019).

Critical in our simulations is the existence of a stable spiral wave within the heterogeneous region. As can be expected, this stable spiral wave is only present if the heterogeneity is sufficiently large. For the LR model, for example, we have verified that reducing the radius of the heterogeneity from R = 0.75 cm to R = 0.65 cm no longer supported a single stable spiral wave, resulting in spiral wave breakup in the entire computational domain. We should also point out that, although we did not observe this in our simulations, it is well known that inexcitable objects can anchor spiral waves (Zou et al., 1993), which would make spontaneous termination more complicated. In addition, our ablation region was modeled as an inexcitable object. Therefore, more complex behavior, such as dynamic anchoring of spiral waves (Vandersickel et al., 2018) or tip trajectories that enter the heterogeneity (Defauw et al., 2013) were not observed either.

Transient chaotic dynamics in spatially extended systems, including the Fenton-Karma model, has also been addressed in a recent study, which revealed the existence of a so-called terminal transient phase immediately prior to the termination of chaotic dynamics (Lilienkamp and Parlitz, 2018). It was shown that during this terminal transient phase, perturbations applied to the system can have a significant effect on the transient lifetime of the termination even though this impeding termination is not evident from the dynamics of commonly observed variables, including the number of spiral wave tips. This observation was used in a subsequent study to demonstrate that chaotic dynamics in spatially extended systems can be terminated using a low number of localized perturbations (Lilienkamp and Parlitz, 2020).

The variable time delay of removal from the source predicted by the simulations was confirmed in direct clinical observations. Ablation that targeted only the locations of spiral wave sources enabled AF to persist for a variable amount of time, which could last up to minutes. This is consistent with other studies, which reported delays between ablation and the termination of AF up to days (Sommer et al., 2016). Taken together, our computational and clinical results suggest a scenario in which spiral wave sources and tissue heterogeneities play a critical role in the maintenance of AF.

Our finding that the spatio-temporal organization preceding termination did not significantly increase suggests that sources were successfully removed and that the remaining tissue activation is a stochastic process that can be described by birth and death events of spiral waves. Consequently, the time to termination after the removal of localized sources is a stochastic quantity and the mean termination time can only be determined as an average value. This mean termination time will depend on both electrophysiological properties of the tissue, including excitability and conduction velocity. Furthermore, it will also depend on anatomical parameters, including the size of the atria (Vidmar and Rappel, 2019) (Supplementary Figure S3). The latter observation can potentially explain clinical data that patients with larger atria are less likely to show abrupt AF termination after source ablation even if long-term outcome is ultimately favorable (Haissaguerre et al., 2014).

Our findings indicate that spiral wave sources are spatially associated with the heterogeneities and, once these sources are removed, AF will perpetuate through spiral wave dynamics, which has a finite probability of termination. Thus, our results also suggest that the elimination of spiral wave sources may be one treatment path for AF. This may explain why limited ablation at sources can terminate even advanced AF (Narayan et al., 2012b), how missing sources by ablation can thus result in recurrence (Baykaner et al., 2018), and how extensive ablation may not terminate AF in such patients (Narayan et al., 2012b; Verma et al., 2014). Other potential therapeutic options involve removing tissue heterogeneities and thus limiting the impact of any residual or emerging sources. Future studies are required to determine how atrial remodeling or other pathophysiology governs this stochastic creation and annihilation of spiral waves.

### Limitations

First, the computational simulations were carried out in flat, 2D geometries. We are currently developing computational methods to study spiral wave dynamics in patient-specific 3D geometries but do not expect that the qualitative results will be affected. Secondly, this is a relatively small cohort of patients and larger studies may enable more accurate determinations of termination time following targeted ablation, which could be extended to patients with persistent AF and even greater tissue heterogeneity. Termination of clinical AF may not predict long-term freedom from AF recurrence, but the focus of our study was the impact of acute perturbation of spiral wave dynamics. As stated, early AF patients were included in this study to enrich the number of cases of acute AF termination, and future studies should address patients with more advanced AF (persistent AF).

## Data Availability

The raw data supporting the conclusion of this article will be made available by the authors, without undue reservation.
