# Supplementary material for: Stochastic Termination of Spiral Wave Dynamics in Cardiac Tissue
Source: Front Netw Physiol. 2022 Jan 26;2:809532. doi: 10.3389/fnetp.2022.809532 (PMC9524168; doi:10.3389/fnetp.2022.809532)
Supplement: Supplementary file 1 [file DataSheet1.docx]

**Supplementary Information for:**

**Stochastic termination of spiral wave dynamics in cardiac tissue**

Sanjiv M. Narayan, David E. Krummen, Tina Baykaner, Junaid Zaman,

Alan Donsky, Vijay Swarup, John M. Miller and Wouter-Jan Rappel

*Computational Studies*

Computational models of 2-dimensional sheets were carried out using the monodomain equation:

$$\frac{dV}{dt}=D\nabla^{2}V-\frac{I_{ion}}{C_{m}}$$

Here, V is the membrane voltage, *C_m_=1μF/cm^2^* represents the membrane capacitance, *D* is the diffusion constant and *I_ion_* represents the membrane currents. The electrophysological model used in the main text was based on Luo-Rudy membrane kinetics ^1^, modified for atrial tissue ^2, 3^. Specifically, the channel conductances G_Na_, G_K_ and G_si_ were set to 16, 0.423 and 0.053 mS/cm^2^, respectively, which resulted in spontaneous spiral wave break up. The conduction velocity was found to be approximately 40 cm/s with an APD_90_ of roughly 120ms, determined using a basic cycle length of 1000ms..

Eq. 1 was simulated on square grids of variable sizes with non-conducting boundaries using a standard finite difference scheme with a spatial discretization of 0.025 cm and a time step of 0.1 ms. A central zone of radius *R* was introduced as a spatial heterogeneity in which one of the model parameters, G_si_, was altered and set to G_si_=0. This ensured that a single spiral wave that was trapped inside the zone would indefinitely continue to rotate within the zone, with a rotation period of approximately 55ms and a wavelength of 1.6cm. This is illustrated in Fig. S2, where we have plotted the tip trajectory of the spiral wave trapped within the spatial heterogeneity, indicated by the red circle.

Ablation lesions were modeled as circular regions with a radius *R_abl_*=0.75cm in which conductivity was set to zero, rendering the tissue inexcitable. In the former, we implemented the phase-field method which is well suited to model appropriate boundary conditions on curved interfaces while using a rectangular finite difference grid ^4, 5^.

The number of spiral wave tips was computed at a temporal discretization of 1ms. From this, we computed the transition rates between two different tip numbers *n*. These rates were used to construct a transition matrix *Q* for all transient states *n > 0*, with elements *Q_ij_* representing the probability of transitioning from state *i* to state *j*. Due to the high temporal resolution, rates that increased or decreased the number of tips by more than 2 were exceedingly rare. As a consequence, the resulting transition matrix is a pentadiagonal matrix. Using the transition matrix, one can compute first passage statistics of birth-death systems with an absorbing state at n = 0 from simple linear algebra ^6^. The probability of reaching state *j* from state *i* in t steps can be shown to be given by the ijth entry of *Q^t^*. Summing this over all time results in the so-called fundamental matrix *N = I + Q + Q^2^ + ... = (I − Q)^-1^*, where *I* is the identity matrix. Each element of the fundamental matrix *N_ij_* represents the mean duration the system will spend in state *j* given an initial state *i*, which can be used to determine the quasi-stationary distribution (Fig 2 and Fig. S4). Moreover, the mean time to extinction is given by $N\vec{e}$, where $\vec{e}$ is a column vector of ones (Table 1 and Table S2).

*Fenton-Karma model results*

Additional numerical simulations were performed simulations using the Fenton-Karma (FK) model ^7, 8^. Parameter values for the FK model were chosen from set #8 of Ref 4 with D= *D*=0.0005 cm^2^/ms. For these parameter values, the conduction velocity was found to be approximately 20 cm/s with an APD_90_ of roughly 180ms, determined using a basic cycle length of 1000ms. A spatial heterogeneity with radius *R*=0.625cm was introduced by changing the parameter τ_r_ from τ_r_=33.25 to τ_r_=20. In these simulations, a single stable spiral, with a period of 80ms and a wavelength of 0.74cm, was constrained to the spatial heterogeneity and was surrounded by spiral wave breakup.

The removal of the heterogeneity zone through virtual ablation resulted in spiral wave dynamics with a finite probability of termination. The mean termination time was computed using 400 independent initial conditions, except for the largest domain size (56.25 cm^2^) where it was computed using 100 independent initial conditions. The distribution of termination times for different domain sizes is shown in Fig. S4A-C on a linear-log scale. The dashed lines represent exponential fits to the distributions. The resulting rates are presented in Supplementary Table S2, except for the largest domain size. For that domain size, we only simulated 100 termination events, making it not feasible to produce an accurate fit to the distribution. The rates agree well with the results from the direct simulations.

The birth and death rates for the spiral wave dynamics of this model, rescaled by the total length of the non-conduction boundary and the total area of the computational domain, are shown as a function of the tip density in Fig. S4D-E. As for the Luo-Rudy model, the rescaled rates collapse to a single curve (cf. Fig. 1). The transition matrix constructed using these rates can be used to compute the quasi-stationary probability distribution. This distribution agrees well with the distribution computed from the direct simulations (Fig. S4F). The transition matrix can also be used to compute the mean termination time.

*Clinical mapping*

The 31 patients in this study had episodes that were self-terminated within 7 days, despite one or more anti-arrhythmic drugs, with no prior AF ablation and left atrial diameters <60 mm in each axis to facilitate good coverage of the multi-electrode array. Population details are presented in Supplementary Table S1. Electrical signals were recorded from baskets containing 64 electrodes (Constellation, Boston Scientific, MA) that were placed in each atrium. Their locations were recorded relative to atrial geometry obtained from clinical mapping systems (NavX, St Jude Medical, MN) (Fig. S1). The electrode array was repositioned to record in multiple positions to cover the majority (>70%) of both atria ^9^ with sufficient spatial resolution to resolve electrical circuits predicted and observed in optically mapped ^10^ human AF. Electrode locations were registered within patient-specific atrial geometry and electrograms were analyzed for activation onset times ^11^ (Fig. 5). Electrograms were analyzed in blocks of 4 s to determine activation times and construct phase maps, as previously described ^12^.

*Wavefront Field (WFF)*

Activation times were used to determine an activation front, as described before ^13^. In brief, time is measured in bins of 10 ms and for each electrode *i* we define a variable φ_i_(t) which takes on the value 1 if an activation occurs within the time bin and 0 otherwise. Smooth spatial activation fronts are computed by convolving φ_i_ with a Gaussian Kernel. From these activation fronts we determine a WFF describing conduction propagation by computing and matching spatial gradients. By computing the vorticity of this flow field we can quantify the intensity of local rotational activity across the mapped domain (Fig. 3, main manuscript) ^13^.

*Phase Synchrony*

In order to compute the level of phase synchrony between electrodes we extend an earlier analysis ^14^ in which the activation times from the clinical recordings are converted into phase-time information. The phase of each electrode increases by 2π between each subsequent activation, with linear interpolation determining phase between activations. The synchronization number γ, defined as the amplitude of the first Fourier mode of the cyclic relative phase distribution ^15^, is computed between pairs of electrodes and quantifies the extent to which that pair displays synchronous activation sequences. The maximum value of γ (γ=1) corresponds to pairs that are perfectly in phase (i.e., phase locked) while pairs that are asynchronous will have γ=0.

To determine a global measure of spatio-temporal organization for a given episode in a given chamber (LA or RA), we compute the mean synchronization number ‹ γ › across all pairs of electrodes. This analysis is computed on three seconds near the middle of each recording block, and any epoch with less than half of the electrodes recording viable data is discarded. To ensure sufficient phase information exists when computing the synchronization number, it is required that at least 1.5 seconds of relative phase is present between the two electrodes.

*Clinical details and outcome*

We monitored patients for 1 year after the procedure using implanted loop recorders in 64.5% of patients. Of n=31 patients, n=26 (83.9%) had no recurrent AF, and n=21 (67.7%) had no recurrent AF nor atrial tachycardia during 1 year after a single procedure (without pulmonary vein isolation), off all anti-arrhythmic medication. An event-free survival plot, made by the Kaplan-Meier method, is presented in Fig. S5. The results compare favorably with the single-procedure success of ~65% from PVI in recent trials ^16^, which involves ablation of significantly more tissue.

References

1. Luo C-h and Rudy Y. A model of the ventricular cardiac action potential. Depolarization, repolarization, and their interaction. *Circulation research*. 1991;68:1501-1526.

2. Virag N, Jacquemet V, Henriquez CS, Zozor S, Blanc O, Vesin JM, Pruvot E and Kappenberger L. Study of atrial arrhythmias in a computer model based on magnetic resonance images of human atria. *Chaos*. 2002;12:754-763.

3. Li D, Zhang L, Kneller J and Nattel S. Potential ionic mechanism for repolarization differences between canine right and left atrium. *Circulation research*. 2001;88:1168-1175.

4. Kockelkoren J, Levine H and Rappel W-J. Computational approach for modeling intra-and extracellular dynamics. *Physical Review E*. 2003;68:037702.

5. Fenton FH, Cherry EM, Karma A and Rappel W-J. Modeling wave propagation in realistic heart geometries using the phase-field method. *Chaos*. 2005;15:013502 (11 pages).

6. Newman M. *Networks*: Oxford university press; 2018.

7. Fenton F and Karma A. Vortex dynamics in three-dimensional continuous myocardium with fiber rotation: Filament instability and fibrillation. *Chaos*. 1998;8:20-47.

8. Fenton FH, Cherry EM, Hastings HM and Evans SJ. Multiple mechanisms of spiral wave breakup in a model of cardiac electrical activity. *Chaos*. 2002;12:852-892.

9. Narayan SM, Krummen DE and Rappel W-J. Clinical Mapping Approach to Identify Rotors and Focal Beats in Human Atrial Fibrillation. *J Cardiovasc Electrophysiol*. 2012;23:447-454.

10. Hansen BJ, Zhao J, Li N, Zolotarev A, Zakharkin S, Wang Y, Atwal J, Kalyanasundaram A, Abudulwahed SH, Helfrich KM, Bratasz A, Powell KA, Whitson B, Mohler PJ, Janssen PML, Simonetti OP, Hummel JD and Fedorov VV. Human Atrial Fibrillation Drivers Correlated With Integrated Functional and Structural Imaging to Benefit Clinical Mapping. *JACC: Clinical Electrophysiology*. 2018.

11. Kuklik P, Zeemering S, Maesen B, Maessen J, Crijns HJ, Verheule S, Ganesan AN and Schotten U. Reconstruction of instantaneous phase of unipolar atrial contact electrogram using a concept of sinusoidal recomposition and Hilbert transform. *IEEE transactions on bio-medical engineering*. 2015;62:296-302.

12. Narayan SM, Krummen DE and Rappel WJ. Clinical mapping approach to diagnose electrical rotors and focal impulse sources for human atrial fibrillation. *J Cardiovasc Electrophysiol*. 2012;23:447-54.

13. Vidmar D, Narayan SM, Krummen DE and Rappel W-J. Determining conduction patterns on a sparse electrode grid: Implications for the analysis of clinical arrhythmias. *Phys Rev E*. 2016;94:050401.

14. Vidmar D, Narayan SM and Rappel WJ. Phase synchrony reveals organization in human atrial fibrillation. *American journal of physiology Heart and circulatory physiology*. 2015;309:H2118-26.

15. Rosenblum M, Pikovsky A, Kurths J, Schäfer C and Tass PA. Phase synchronization: from theory to data analysis. *Handbook of biological physics*. 2001;4:279-321.

16. Kuck KH, Brugada J, Furnkranz A, Metzner A, Ouyang F, Chun KR, Elvan A, Arentz T, Bestehorn K, Pocock SJ, Albenque JP, Tondo C, Fire and Investigators ICE. Cryoballoon or Radiofrequency Ablation for Paroxysmal Atrial Fibrillation. *N Engl J Med*. 2016;74:2235-45.

Supplementary Table 1: Patient details

| **Characteristic** |  |
| --- | --- |
| Gender (M/F) | 31 (28/3) |
| Age /years | 59±12 (23-78) |
| History of AF /years | 3.8±3.4 (0.3-15.3) |
| Largest LA diameter, Preprocedural /mm* | 44±7 (36-62) |
| LVEF /% (range) | 52±17 (20-75) |
| CHADS2 Score | 1.5±1.0 |
| No. With CHADS2 ≥2 | 38.7% (12) |
| Hypertension / % (n) | 74.2% (23) |
| Coronary Disease / % (n) | 32.3% (10) |
| Previously ineffective anti-arrhythmic medications | 1.3±0.7 |
| Number of prior ablations | 0 |

Supplemental Table 2: Termination times computed using simulations of the Fenton Karma model, obtained from fits to the distribution, and determined using the transition rate matrix.

| Domain size (cm^2^) | τ (simulations) (s) | λ (Poisson) (s) | T_av_ (matrix) (s) |
| --- | --- | --- | --- |
| 25 | 4.1 | 4.6 | 4.1 |
| 31.6 | 8.0 | 8.6 | 8.1 |
| 39 | 25 | 27 | 25 |
| 56.25 | 287 | - | 288 |

**
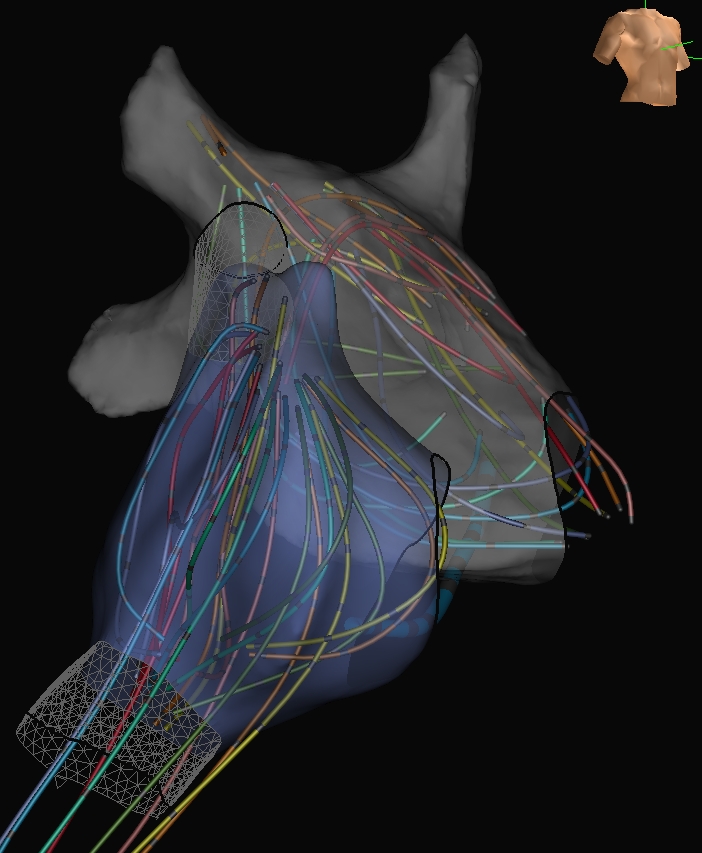
**

**Supplemental Figure 1**

Multipolar basket catheters in both human atria, deployed to maximize atrial coverage. The colored lines show the basket splines, containing the electrodes.

**Supplementary Figure 2**

Computational domain of size 5cmx5cm containing a tissue heterogeneity of *R*=0.75cm (red line). The black line represents the spiral wave tip trajectory of the single spiral wave of the Luo-Rudy model trapped in the heterogeneous zone, further shown in the magnified view of the inset. Scale bar: 1cm.

**Supplementary Figure 3**

The mean termination time τ as a function of the net area, following ablation of a small heterogeneous region that captured a stable spiral wave source (see Fig. 1 main text and Fig. S2) for the Luo-Rudy model (A) and the Fenton-Karma model (B). The dashed lines are an exponential fit $ae^{(cA_{net})}$ with a coefficient of *c*=0.039/cm^2^ (A) and *c*=0.14/cm^2^ (B).

**Supplementary Figure 4**

**Computational results for the Fenton-Karma model.** **A-C**, Distribution of termination times, computed using 400 independent simulations, for a domain size of 25 cm^2^ (A), 31.6 cm^2^ (B), and 39 cm^2^ (C). The dashed line is an exponential fit to the distribution (resulting rates are provided in Table S2). **D**, The W_±1_ rates, normalized by the perimeter of the domain, as a function of the density of tips, *q = n/A_net_*. **E,** The W_±2_ rates, normalized by the net area of the domain, as a function of the density of tips. **F,** The quasi-stationary distribution for a domain size of 39 cm^2^ computed using the simulations (lines) and computed using the transition matrix (symbols).

**Supplemental Figure 5**

Cumulative freedom from AF (solid line) and from all arrhythmias (dashed line) after a single AF source ablation procedure. Freedom from AF was 83.9% at 365 days and freedom from all arrhythmias was 67.7% at 1 year.


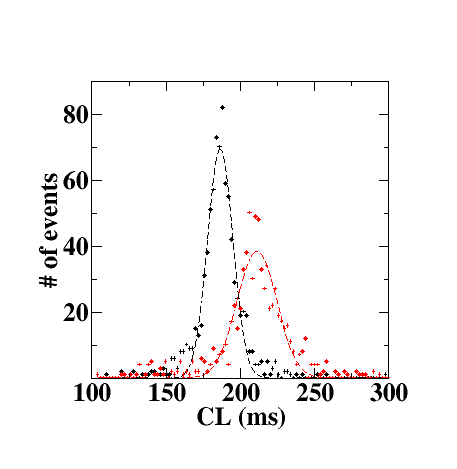


**Supplemental Figure 6**

Cycle length (CL) distributions (symbols), shown here along with a Gaussian fit (dashed lines), for patients immediately preceding termination (black distribution: 45 year old male, red distribution: 59 year old male).
